# Probiotics and Antibiotics: From Empirical Practice to a Biological Rationale for Targeted Choice During Antibiotic Therapy

**DOI:** 10.3390/microorganisms14040763

**Published:** 2026-03-27

**Authors:** Mariarosaria Matera, Valentina Biagioli, Stefano Leo, Lorenzo Drago

**Affiliations:** 1 Department of Pediatric Emergencies, Misericordia Hospital, 58100 Grosseto, Italy; 2 Department of Neurosciences, Rehabilitation, Ophthalmology, Genetics, Maternal and Child Health, University of Genoa, 16126 Genoa, Italy; valentina.biagioli@edu.unige.it; 3 European Biomedical Research Institute of Salerno, 84125 Salerno, Italy; 4 UOC Laboratory of Clinical Medicine with Specialized Areas, IRCCS MultiMedica Hospital, 20099 Milan, Italy; lorenzo.drago@unimi.it; 5 Clinical Microbiology and Microbiome Laboratory, Department of Biomedical Sciences for Health, University of Milan, 20133 Milan, Italy

**Keywords:** gut microbiota, antibiotic therapy, probiotics, microbial ecology, dysbiosis, microbiome resilience, host–microbe interactions

## Abstract

Antibiotic therapy represents one of the strongest ecological perturbations of the human gut microbiota, inducing rapid and often prolonged alterations in community structure, metabolic activity, and functional resilience. While the use of probiotics to mitigate antibiotic-associated dysbiosis is widely adopted in clinical practice, probiotic selection is still largely empirical and insufficiently grounded in biological compatibility with specific antibiotic pressures. In this conceptual review, antibiotics are reframed not merely as antimicrobial agents, but as ecological forces that shape microbial survival, quiescence, and recolonization dynamics. We propose a biologically informed framework that distinguishes genetic antibiotic resistance from functional or ecological insensitivity, highlighting how microbial traits, such as the absence or inaccessibility of the antibiotic target, metabolic state, sporulation, and cellular architecture, influence the persistence of probiotics during antibiotic exposure. By integrating the mechanisms of action of antibiotics with key physiological and structural features of probiotic microorganisms, we develop a conceptual framework aimed at rationalizing the compatibility of probiotics and antibiotics. This framework does not imply clinical efficacy but provides an interpretative tool to guide hypothesis generation, experimental validation, and the design of future targeted probiotic strategies. A more ecologically grounded approach to probiotic selection may ultimately improve microbiota support during antibiotic therapy and advance personalized microbiome modulation.

## 1. Introduction

Antibiotic therapy is a major factor disrupting the intestinal microbiota, with effects detectable within a few days of treatment and persisting for weeks or months [1,2].

Antibiotic dysbiosis is a dynamic ecological trajectory characterized by a complex alteration of the intestinal microbial ecosystem, affecting composition, relative abundance, and functionality [3]. Each antibiotic class exerts a specific selective pressure, modulated by the spectrum of activity, dose, duration, and route of administration, as well as by the intrinsic resilience of the host microbiota, which is influenced by age, health status, diet, and lifestyle [4]. Across experimental and clinical studies, antibiotic exposure has been associated with measurable reductions in bacterial richness and diversity, accompanied by marked restructuring of community composition. In some settings, antibiotic-induced dysbiosis has also been linked to paradoxical increases in total microbial load and shifts in the relative abundance of Gram-positive and Gram-negative taxa, although the magnitude and direction of these changes vary widely depending on antibiotic class, exposure characteristics, host context, and baseline microbiota configuration [5,6,7]. Dysbiosis can persist for weeks or months, with recovery varying depending on the characteristics of the antibiotic therapy administered and the host [8]. Notably, the observed effects of antibiotics on microbial diversity and load depend on the analytical technology employed. Methods such as 16S rRNA gene sequencing, shotgun metagenomics, and quantitative PCR (qPCR) differ in how they quantify microbial abundance (relative in sequencing-based approaches versus absolute in qPCR) and in their taxonomic resolution, particularly in detecting low-abundance or rare species [9].

Beyond the treatment of acute infections, antibiotics are used for prolonged prophylaxis or suppressive purposes, even for months or years, in various selected clinical settings. The most well-established example is the secondary prophylaxis of rheumatic fever, in which continuous penicillin administration reduces the risk of relapse and progression to rheumatic heart disease [10,11]. A similar approach is also applied in recurrent urinary tract infections, both in adults and children, where low-dose antibiotics are administered to reduce the frequency of relapses [11].

Short-term effects of antibiotic treatment include antibiotic-associated diarrhea (AAD), *Clostridioides difficile* colonization, and an increased risk of opportunistic infections and multidrug-resistant organisms (MDROs) [3]. In the long term, persistent dysbiosis has been linked to altered immune, metabolic, neurodevelopment trajectories, increasingly linked to non-communicable diseases [3,4,5].

Probiotic administration during antibiotic therapy has historically been framed as a strategy to counteract antibiotic-associated perturbations of the gut microbiota. This view, however, has largely been shaped by empirical practice, often overlooking the complex interplay between antibiotic pharmacodynamics, microbial ecology, and the intrinsic biological features of the administered strains.

Antibiotics are now recognized not merely as agents of microbial suppression, but as strong ecological forces that reshape community structure and function within the gut ecosystem [12]. Metagenomic analyses consistently demonstrate that even brief antibiotic exposures can profoundly alter microbial diversity, eliminate keystone taxa, and generate new ecological niches that may be transient or long-lasting [13,14]. Within such a dynamically restructured ecosystem, probiotic supplementation represents an active ecological intervention rather than a neutral adjunct. Accordingly, antibiotic–probiotic compatibility should be conceptualized beyond simple strain survival and instead interpreted through an ecological lens that considers the alignment between the antibiotic’s mode of action and the physiological, functional, and ecological traits of the introduced microorganism.

In this review, we aim to propose a conceptual framework that relates the type of antibiotic, its mechanism of action, and probiotic biological characteristics.

## 2. Materials and Methods

### 2.1. Search Strategy

This article was developed as a narrative and conceptual review aimed at integrating current knowledge on antibiotic mechanisms of action, their ecological impact on the gut microbiota, and the biological traits of probiotic microorganisms that may influence their persistence under antibiotic pressure. A structured but non-systematic literature search was conducted using PubMed, Scopus, and Web of Science to identify relevant publications addressing antibiotic–microbiota interactions and probiotic biology. The search primarily covered articles published between 2000 and 2026, although earlier seminal studies were included when considered essential for understanding fundamental biological mechanisms. Search terms included combinations of antibiotics, gut microbiota, ecological impact, probiotics, antibiotic resistance, antibiotic tolerance, sporulation, and microbial metabolism.

### 2.2. Inclusion and Exclusion Criteria

Eligible publications included experimental studies, mechanistic microbiology studies, narrative and systematic reviews, and conceptual papers addressing the biological and ecological consequences of antibiotic exposure and their potential implications for probiotic function. Studies were selected based on their relevance to the conceptual objectives of the review and their contribution to understanding the interactions between antibiotics, microbial physiology, and gut microbial ecology.

To improve transparency of the search process, additional methodological details are provided in the Appendix A.

### 2.3. Figure Production

**Figure 1:**
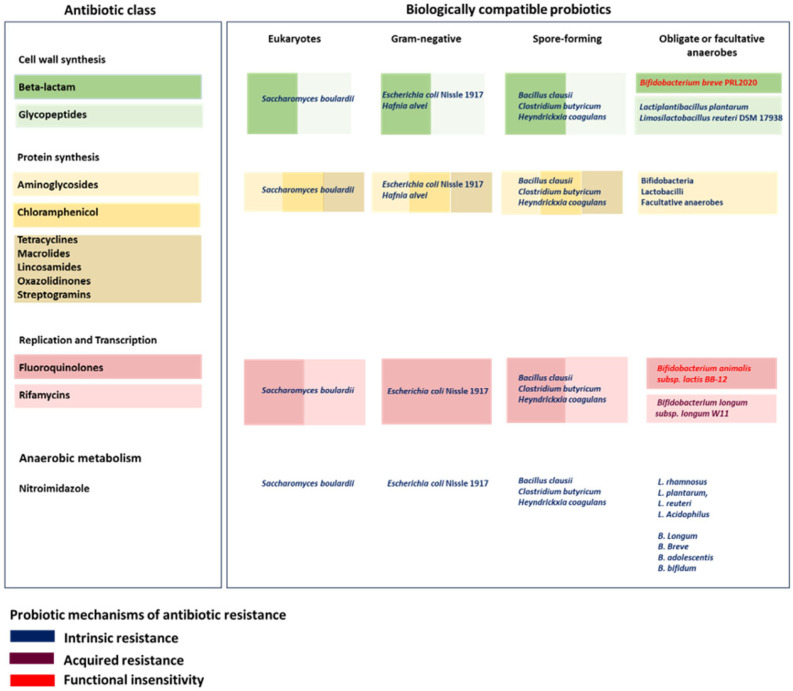
Conceptual framework of probiotic-antibiotic biological compatibility. This grid integrates antibiotic mechanisms of action with key biological traits of probiotic microorganisms to illustrate theoretical patterns of compatibility under antibiotic pressure. Compatibility is interpreted in biological and physiological terms, distinguishing intrinsic resistance and functional insensitivity from acquired resistance. The framework is conceptual and does not imply clinical efficacy or therapeutic recommendations.

Graphical abstract was created in part with BioRender (https://BioRender.com) and further edited using Microsoft PowerPoint and Inkscape (Version 1.4.3) whereas Figure 1 was entirely designed with using Microsoft PowerPoint and Inkscape.

## 3. Key Concepts and Operational Definitions for Probiotics

This chapter introduces and defines core concepts, establishing a shared language that enables accurate interpretation of the literature and supports a conceptual framework for probiotic selection during antibiotic therapy.

### 3.1. Probiotic: Regulatory vs. Biological Definition

From a regulatory perspective, probiotics are defined as “live microorganisms which, when administered in adequate amounts, confer a health benefit on the host” [15]. While suitable for regulatory purposes, this definition does not address the biological requirements necessary for probiotic functionality during antibiotic coadministration, particularly the ability of a strain to survive antibiotic-driven selective pressure [15]. From a biological and ecological standpoint, a probiotic intended for use during antibiotic therapy may be expected to exhibit specific functional properties, including viability under exposure to the relevant antibiotic, ability to colonize, and interact with the resident microbiota. These functional traits are supported by mechanistic studies, although clinical impact may vary with host and context.

### 3.2. Resistance to Antibiotics: Conceptual Clarifications and Application to Probiotics

Conceptually, we should distinguish:

#### 3.2.1. Intrinsic Resistance (IR)

It is an innate, species- or genus-level property resulting from stable, genetically encoded characteristics that inherently prevent antibiotic activity. IR is due to:

(a) Absence of a molecular target, when the antibiotic target is not present in the microorganisms.
(b) Absence of drug activation systems, in the case of prodrugs requiring metabolic activation, which cannot exert antibacterial activity in microorganisms lacking the necessary activation pathways.
(c) Structural or metabolic barriers, including reduced drug permeability due to cell wall or membrane features, limited porin expression, to metabolic state incompatible with antibiotic action, such as growth arrest or functional quiescence. This may occur under specific metabolic conditions, such as insufficient redox potential required for the activity of certain antibiotics [16].

#### 3.2.2. Acquired Resistance (AR)

Bacteria develop resistance to drugs to which they were once susceptible. AR can be subdivided into:

(a) Non-transferable chromosomal resistance: resulting from genes encoded in the bacterial chromosome.
(b) Transferable acquired resistance: mediated by mobile genetic elements that may be horizontally transferred to other bacteria.

#### 3.2.3. Functional Insentivity (FI)

Functional insensitivity (FI) represents a concept distinct from antibiotic resistance, tolerance [13], and persistence. While tolerance refers to the slowed killing of bacteria without tangible increases in MIC and persistence describes dormant subpopulations that survive antibiotic exposure, FI arises when intrinsic physiological or structural traits limit the effective interaction between an antibiotic and its cellular targets [13]. Unlike classical resistance, FI is not mediated by transferable resistance genes but by strain-specific features such as cell envelope architecture, exopolysaccharide production, metabolic state, or reduced growth that diminish antibiotic impact. Consequently, FI should be considered a strain-dependent property, as closely related strains may differ substantially in traits influencing antibiotic interaction.

From an experimental point of view, distinguishing resistance, tolerance, persistence, and FI requires complementary approaches: MIC testing identifies growth inhibition, whereas time-kill assays capture differences in bacterial survival dynamics (Table 1). Within this framework, FI would manifest as minimal antibiotic effect despite the absence of resistance determinants. Importantly, distinguishing non-transferable mechanisms such as intrinsic resistance or FI from acquired resistance is essential for probiotic safety assessment, as only the latter poses significant risks related to antimicrobial resistance dissemination.

### 3.3. Viability, Functionality, and Colonization

Viability and colonization represent distinct biological concepts in probiotic research. A probiotic microorganism may exert biological activity during gastrointestinal transit without establishing long-term colonization, provided that viability and metabolic functionality are preserved [17]. Conversely, stable colonization and persistence depend on both intrinsic strain characteristics and host-related factors [17,18,19]. Failure to distinguish between viability, functionality, and colonization in clinical studies contributes to heterogeneous interpretations of the results and limits the possibility of defining rational combination strategies between probiotics and antibiotics.

## 4. Antibiotics and Compatible Probiotics

In this section, antibiotic mechanisms of action will be discussed according to three major functional categories (cell wall synthesis inhibition, protein synthesis inhibition, and interference with nucleic acid metabolism), with a focus on probiotics that could be biologically compatible.

### 4.1. Antibiotics That Inhibit Cell Wall Synthesis: β-Lactams and Glycopeptides

#### 4.1.1. Mechanisms of Action

β-lactams and glycopeptides share a common biological target: cell wall synthesis, which is essential for maintaining bacterial structural integrity and protecting against osmotic lysis, although they act through distinct molecular mechanisms [20,21].

β-lactams, including penicillins, cephalosporins, carbapenems, and monobactams, exert their antibacterial activity by inhibiting Penicillin Binding Proteins (PBPs), enzymes involved in the terminal steps of peptidoglycan synthesis. A crucial aspect from a biological point of view is that the activity of β-lactams is also strictly dependent on the metabolic state of the target microorganisms. These antibiotics are highly effective against actively growing microorganisms, but show reduced efficacy against dormant, spore-forming, or slow-metabolizing bacteria [22,23].

Glycopeptides, such as vancomycin and teicoplanin, act through a different mechanism. They do not interact directly with PBPs but bind directly to the D-Ala–D-Ala terminus of peptidoglycan precursors; this bond creates a steric hindrance that impedes both transglycosylation and transpeptidation [24,25]. These antibiotics have large molecular sizes, which prevent them from crossing the outer membrane of Gram-negative bacteria. The outer membrane represents a physical barrier to drug access to the peptidoglycan, so glycopeptides are active almost exclusively against Gram-positive bacteria and ineffective against Gram-negative bacteria [24].

#### 4.1.2. Probiotics Are Biologically Compatible with Cell-Wall-Acting Antibiotics

**Eukaryotic Microorganisms Without Antibiotic Target**

The yeast *Saccharomyces boulardii* represents the paradigmatic example of a probiotic completely devoid of an antibiotic target, being a eukaryote with a cell wall composed of glucans and chitin. Consequently, it is intrinsically insensitive to β-lactams and glycopeptides, maintaining viability and functional activity during antibiotic therapy [1,2,3,4,5,6,7,8,9,10,11,12,13,14,15,16,17,18,19,20,21,22,23,24,25].

**Bacteria with Limited Access to Peptidoglycan**

Alongside strains with documented intrinsic resistance, a second conceptual category can be included: microorganisms whose cellular architecture limits functional access to the antibiotic target.

In Gram-negative bacteria, the interaction between antibiotics and microbial cells is strongly influenced by the structural organization of the cell envelope, particularly the presence of an outer membrane that acts as a permeability barrier. This outer membrane represents a major determinant of intrinsic antibiotic susceptibility, as it limits the diffusion of many antimicrobial molecules into the periplasmic space [26].

In some cases, the antibiotic is effectively excluded because its physicochemical properties prevent penetration through the outer membrane. This is the case for glycopeptides such as vancomycin, which are generally inactive against Gram-negative bacteria due to their large molecular size and inability to cross the outer membrane barrier [27]. In other cases, antibiotic entry depends on specific permeability pathways, particularly outer membrane porins. Many hydrophilic antibiotics, including several β-lactams, reach the periplasmic space through porin channels that allow the diffusion of small hydrophilic molecules [28].

Alterations in porin expression or structure can significantly reduce antibiotic influx and therefore contribute to decreased susceptibility or resistance [28]. Finally, additional mechanisms may further modulate intracellular antibiotic concentration after entry into the cell. These include active efflux systems and enzymatic inactivation mechanisms, such as β-lactamases, which together contribute to the complex phenotype of antimicrobial resistance [29].

In conclusion, susceptibility to cell wall-acting antibiotics in these microorganisms depends largely on outer membrane permeability, porin expression, and target affinity [30].

In this context, selected Gram-negative probiotic strains used in humans, such as *Escherichia coli* Nissle 1917 and *Hafnia alvei* HA4597^®^, may be conceptually viewed as microorganisms with a functionally shielded antibiotic target, rather than as resistant strains in the classical sense [28,29].

**The case of vancomycin and some lactobacilli, such as *Lactiplantibacillus plantarum* and *Limosilactobacillus reuteri* DSM 17938** **.**

Vancomycin deserves a separate discussion, as its target is the D-Ala-D-Ala termini of peptidoglycan precursors. Some lactobacilli, including *Lactiplantibacillus plantarum* and *Limosilactobacillus reuteri* DSM 17938, possess an alternative structure of the muramic pentapeptide D-alanine D-lactate (D-Ala-D-Lac) instead of the standard D-Ala-D-Ala. This drastically reduces the antibiotic’s affinity for the target site. This resistance does not depend on transferable genes, but on species-specific enzymes, such as an atypical D-Ala-D -Ala ligase and a VanX-like dipeptidase, which promote the synthesis of D-Ala-D-Lac depsipeptides and degrade any residual D-Ala-D-Ala, conferring a high-level intrinsic vancomycin-resistant phenotype [30,31]. This characteristic determines intrinsic, species-specific, and non-transferable resistance, conceptually distinct from the acquired resistance mechanisms observed, for example, in enterococcal pathogens.

**Spore-Forming Strains**

Spore-forming probiotics, including *Clostridium butyricum*, *Bacillus clausii*, and *Heyndrickxia coagulans* (formerly *Bacillus coagulans*), exhibit a unique compatibility with β-lactams. Under antibiotic pressure, these organisms enter a metabolically quiescent spore state that is intrinsically refractory to antibiotics targeting dividing cells.

Although butyrate production may be reduced in a vegetative cell, *C. butyricum* spores can survive antibiotic treatment, potentially supporting their ecological function and aiding in the recovery of gut metabolic activity [32].

***Bifidobacterium breve* PRL2020 as an Example of Functional Insensitivity to β-Lactam Antibiotics**

Although most *Bifidobacterium breve* strains are highly sensitive to β-lactam antibiotics like amoxicillin and amoxicillin-clavulanic acid, *B. breve* PRL2020 shows unusually high MIC values [33]. In a large screening of bifidobacterial isolates, the vast majority (≈98%) were inhibited at much lower concentrations; only a few, including PRL2020, showed marked insensitivity, indicating this is a specific characteristic of this strain rather than a species-wide trait [33]. Whole-genome analysis, conducted according to EFSA criteria and based on querying CARD and RGI databases, excluded the presence of antibiotic resistance genes associated with mobile or transferable genetic elements [14,15,16,17,18,19,20,21,22,23,24,25,26,27,28,29,30,31,32,34,35]. The reduced susceptibility to β-lactam antibiotics observed in *B. breve* PRL2020, in the absence of β-lactamase genes or acquired resistance determinants, is consistent with a functional insensitivity related to cell surface characteristics. Specifically, EPS production, altered cell wall permeability, and modulation of intracellular transport systems appear to contribute to the reduced susceptibility to amoxicillin–clavulanate. This profile confers ecological safety and functional stability during therapy with β-lactam/β-lactamase inhibitor combinations, supporting the rationale for the use of this strain in microbiota-support strategies under defined antibiotic pressure.

#### 4.1.3. Conceptual Synthesis

Antibiotics that inhibit cell wall synthesis exert their bactericidal effects primarily on microorganisms actively engaged in peptidoglycan biosynthesis. Consequently, probiotic biological compatibility with these agents is determined not only by the presence of genetic resistance traits, but also by structural, physiological, and metabolic conditions that limit effective drug-target interaction.

Within this framework, compatibility with cell wall-acting antibiotics arises through intrinsic resistance or functional insensitivity, both of which are non-transferable and biologically distinct from acquired resistance mechanisms.

**Intrinsic Resistance**
  - **Absence or modification of the molecular target** (*S. boulardii*, *L. plantarum*, and *L. reuteri* DSM 17938)
  - **Structural inaccessibility of the target** (*shielded target*), as in Gram-negative probiotics (*E. coli Nissle*, *H. alvei*);
  - **Physiological quiescence**, particularly in spore-forming bacteria (*C. butyricum*, *B. clausii*, and *H. coagulans*).

**Functional Insensitivity**
  - **Strain-specific functional insensitivity** (*B. breve* PRL2020 in relation to amoxicillin and AC).

Taken together, these observations support an ecological interpretation in which probiotic survival during exposure to cell wall–acting antibiotics reflects dynamic interactions between antibiotic pharmacodynamics and microbial biology, rather than the presence of fixed resistance phenotypes. This conceptualization provides a biological rationale for informed probiotic selection under defined antibiotic pressures, without implying clinical efficacy or therapeutic recommendations.

An overview of probiotic biological compatibility with cell-wall-acting antibiotics is presented in Figure 1.

### 4.2. Antibiotics That Inhibit Protein Synthesis: Aminoglycosides, Tetracyclines, and Other Ribosomal Classes

#### 4.2.1. Mechanisms of Action

Antibiotics that inhibit protein synthesis act by binding to bacterial ribosomes, thereby blocking the translation of mRNA into proteins essential for cell growth and replication. However, their efficacy is affected by intracellular antibiotic accumulation, metabolic activity, and physiological accessibility of the ribosomal target [36].

The main ribosome-targeting antibiotic classes include aminoglycosides, tetracyclines, macrolides, lincosamides, and chloramphenicol. While aminoglycosides exert a primarily bactericidal activity, most other ribosomal inhibitors are bacteriostatic, with activity preferentially directed toward metabolically active and actively translating bacteria [22].

**Aminoglycosides**

Aminoglycosides (e.g., gentamicin, amikacin) bind to the 30S ribosomal subunit, inducing mRNA misreading and the synthesis of nonfunctional proteins, ultimately leading to bacterial death. Their intracellular uptake is an active, energy-dependent process requiring an intact electrochemical membrane gradient and oxidative metabolism [36,37].

**Tetracyclines**

Tetracyclines (e.g., doxycycline, minocycline) exert a predominantly bacteriostatic effect by binding to the 30S ribosomal subunit and preventing the attachment of aminoacyl-tRNA to the A site. Intracellular accumulation depends on active or facilitated transport systems whose expression varies among species and according to the physiological state of the bacterial cell [38,39].

**Other Ribosomal Antibiotics**

Macrolides, lincosamides, and chloramphenicol act on the 50S ribosomal subunit and primarily affect metabolically active Gram-positive bacteria, including anaerobic commensals involved in short-chain fatty acid (SCFAs) production.

**Chloramphenicol**

Chloramphenicol represents a prototypical inhibitor of bacterial protein synthesis, acting through binding to the peptidyl transferase center of the 50S ribosomal subunit and blocking peptide bond formation [40,41,42]. Its activity is predominantly bacteriostatic.

#### 4.2.2. Probiotics Are Biologically Compatible with Protein Synthesis-Inhibiting Antibiotics

**Eukaryotic Microorganisms Without Antibiotic Targets**

*S. boulardii* possesses eukaryotic ribosomes that are structurally distinct from bacterial ribosomes, rendering it completely insensitive to antibiotics that inhibit bacterial protein synthesis [32].

**Gram-Negative Bacteria Have Limited Intracellular Access**

Some Gram-negative probiotic strains, such as *E. coli* Nissle 1917 and *H. alvei*, possess an outer membrane that restricts antibiotic penetration, limiting intracellular drug accumulation and reducing susceptibility to ribosome-targeting antibiotics [26].

**Microorganisms with Metabolic Quiescence**

Spore-forming probiotic strains, including *B. clausii*, *C. butyricum*, and *H. coagulans*, can enter metabolically quiescent states or form spores under antibiotic pressure. In these conditions, protein synthesis is minimal or absent, depriving both bacteriostatic and bactericidal ribosomal antibiotics of an effective target and allowing probiotic survival and persistence [43].

**The peculiar Case of Aminoglycosides and Anaerobic Probiotics**

Aminoglycosides represent a distinct case among protein synthesis inhibitors. Their uptake requires active transport driven by oxidative metabolism; consequently, penetration is markedly reduced under anaerobic or fermentative conditions, conferring an intrinsic, non-transferable form of functional insensitivity. This explains the limited activity of aminoglycosides not only against quiescent microorganisms and spore-forming probiotics but also against obligate anaerobes such as bifidobacteria and facultative anaerobes or microaerophiles such as lactobacilli, in which the absence of oxidative respiration prevents effective drug internalization [29,36,37,38,39,40,41,42,43,44,45]

**The Peculiar Case of Chloramphenicol and Metabolically Quiescent Probiotics**

Chloramphenicol provides a complementary paradigm among ribosome-targeting antibiotics, as its bacteriostatic activity is strictly dependent on active protein synthesis. Microorganisms characterized by reduced translational activity, slow growth rates, or transient metabolic quiescence exhibit a marked reduction in susceptibility despite the presence of an intact ribosomal target [40,44]. From a probiotic perspective, this translates into a potential biological compatibility with strains that persist in low-metabolic states during antibiotic exposure, including spore-forming bacteria and microorganisms adapted to anaerobic or nutrient-limited niches. In this context, chloramphenicol further supports the concept that probiotic survival during antibiotic therapy may rely more on functional metabolic states than on genetically encoded resistance mechanisms.

#### 4.2.3. Conceptual Synthesis

Antibiotics that inhibit protein synthesis exert their ecological effects primarily on microorganisms that are metabolically active and engaged in active translation. As a consequence, probiotic compatibility with ribosome-targeting antibiotics is determined less by the mere presence of the ribosomal target and more by the conditions governing intracellular antibiotic accumulation and translational activity.

Within this framework, biological compatibility arises through intrinsic resistance or functional insensitivity, both rooted in physiological and structural traits rather than in acquired resistance mechanisms.

**Intrinsic Resistance**
  - **Absence or fundamental modification of the ribosomal target**, as in eukaryotic microorganisms such as *S. boulardii*, whose ribosomes are structurally distinct from bacterial ribosomes and therefore insensitive to antibiotics targeting bacterial protein synthesis.
  - **Lack of oxidative metabolism required for active drug transport:** obligate anaerobes and fermentative bacteria, including *bifidobacteria* and *lactobacilli*, display reduced susceptibility to aminoglycosides due to the absence of oxidative metabolism required for active drug transport.
  - **Restricted intracellular access to the target**: Gram-negative probiotic strains (*E. coli* Nissle 1917, *H. alvei)*.
  - **Physiological quiescence**, particularly in spore-forming bacteria (*C. butyricum*, *B. clausii*, *H. coagulans*), where minimal or absent protein synthesis deprives ribosomal inhibitors of an effective target.

**Functional Insensitivity**

It is exemplified by microorganisms whose metabolic or respiratory characteristics transiently limit antibiotic uptake or activity. Similarly, metabolically quiescent or slow-growing probiotics exhibit reduced susceptibility to bacteriostatic ribosomal inhibitors, such as chloramphenicol, despite the presence of an intact ribosomal target.

An overview of probiotic biological compatibility with protein synthesis-inhibiting antibiotics is presented in Figure 1.

### 4.3. Antibiotics That Interfere with Nucleic Acids: Fluoroquinolones, Rifamycin, and Nitroimidazoles

#### 4.3.1. Mechanisms of Action

Antibiotics that interfere with nucleic acid synthesis target enzymes essential for DNA replication and RNA transcription, leading to irreversible alterations in genomic integrity or gene expression, and consequently growth arrest or cell death. Clinically relevant classes include fluoroquinolones, rifamycins, and nitroimidazoles.

**Fluoroquinolones**

Fluoroquinolones (e.g., ciprofloxacin, levofloxacin) inhibit DNA gyrase (topoisomerase II) and topoisomerase IV, enzymes essential for supercoiling, replication, and segregation of bacterial DNA. Enzyme inhibition leads to the formation of stable enzyme-DNA complexes, resulting in the accumulation of double-strand breaks and cell death [40,41,42,43,44,45,46,47,48]. Their activity is maximal in actively replicating bacteria, while it is significantly reduced in slow-growing, dormant, or spore-forming microorganisms.

**Rifamycins**

Rifamycins (e.g., rifampicin, rifaximin) act by binding to the β-subunit of bacterial DNA-dependent RNA polymerase, blocking transcription initiation and preventing RNA synthesis. As with fluoroquinolones, efficacy is closely related to the bacterial cell’s transcriptional and metabolic status.

**Nitroimidazole**

Nitromidazoles (e.g., metronidazole and tinidazole) are chemically inactive and require intracellular metabolic activation to exert their antimicrobial effects. Activation depends on bacterial redox enzyme system, including nitroreductases, flavoproteins, and pyruvate ferredoxin oxidoreductase (PFOR), which generate toxic nitro radicals capable of inducing DNA damage and chromosomal instability [23,24,25,26,27,28,29,30,31,32,33,34,35,36,37,38,39,40,41,42,43,44,45,46,47,48]. Such antibiotics target primarily obligate anaerobic and metabolically active bacteria [23,24,25,26,27,28,29,30,31,32,33,34,35,36,37,38,39,40,41,42,43,44,45,46,47,48].

#### 4.3.2. Probiotics Biologically Compatible with Protein Synthesis-Inhibiting Antibiotics

**Eukaryotic Microorganisms Without Antibiotic Targets**

*S. boulardii* lacks bacterial DNA gyrases and topoisomerases and possesses a structurally distinct RNA polymerase [1,2,3,4,5,6,7,8,9,10,11,12,13,14,15,16,17,18,19,20,21,22,23,24,25]. Consequently, fluoroquinolones and rifamycins are ineffective, making *S. boulardii* conceptually compatible for co-administration during such antibiotic therapies.

**Spore-Forming and Quiescent Bacteria**

Spore-forming strains, such as *C. butyricum*, *B. clausii*, and *H. coagulans*, undergo phases of markedly reduced transcriptional and replicative activity. Under these conditions, fluoroquinolones and rifamycins are poorly effective, as lethal DNA damage or transcriptional blockade cannot be efficiently fixed [22,23,24,25,26,27,28,29,30,31,32,33,34,35,36,37,38,39,40,41,42,43,44,45,46,47,48,49,50,51].

**Obligate Anaerobes**

Many obligate anaerobes of the gut microbiota display low replication rates and intermittent transcriptional activity.

*C. butyricum*, beyond sporulation, is characterized by reduced functional exposure of molecular targets [52].

Among bifidobacteria, we acknowledge different mechanisms and strategies. *Bifidobacterium longum subsp. longum* W11 exerts an intrinsic resistance to rifamycin through a stable chromosomal (and thus non-transferable) mutations in the *rpoB* gene that reduce RNA polymerase affinity for the drug [51,52]. Other strains, although sensitive to fluoroquinolones in vitro, may enter reversible states of slowed metabolism, i.e., *B. longum*, *B. adolescentis*, and *B. breve* [43,45,46,47,48,49,50,51,52,53,54,55]. *Bifidobacterium animalis* subsp. *lactis* BB-12 adopts stress-induced mechanisms upon fluoroquinolone treatment [56].

**Bacteria with Efflux Pump Systems**

*E. coli* Nissle 1917 and *H. alvei* show variable susceptibility to fluoroquinolones, influenced by porin expression and regulation of transport systems [30]. In fact, *E. coli* Nissle 1917 exhibits AcrAB-TolC-type efflux systems, which further reduce intracellular drug accumulation [57]. Similar detoxification efflux mechanisms have been described in other commensal and in some lactobacilli [58].

#### 4.3.3. Conceptual Synthesis

**Intrinsic Resistance**
  - **Absence of the target:** *S. boulardii.*
  - **Inaccessibility to the target:** spore-forming bacteria (*C. butyricum*, *B. clausii*, and *H. coagulans*).

**Reduced cellular permeability or efflux activity**: Gram-negative strains such as *E. coli* Nissle 1917 and *H. alvei*.

**Non-Transferable Acquired Resistance**
  - **Mutations of the rpoB gene:** *B. longum* subsp. *longum* W11.

**Functional Insensitivity**
  - **Stress-induced mechanisms upon treatment:** *B. animalis* subsp. *lactis* BB-12.

An overview of probiotic biological compatibility with antibiotics that interfere with nucleic acids is presented in Figure 1.

### 4.4. Antibiotics Active on Anaerobic Metabolism

#### 4.4.1. Mechanisms of Action

Antibiotics that selectively interfere with anaerobic metabolism represent a distinct pharmacological class. The prototype of this group is metronidazole, along with other nitroimidazoles, whose antimicrobial activity is strictly dependent on intracellular redox conditions and anaerobic metabolic activity [50]. Unlike antibiotics acting on universal structural or enzymatic targets, nitroimidazoles are prodrugs that require intracellular reductive activation to become biologically active. This process is mediated by a low-redox-potential electron transport system, including ferredoxins and nitroreductases, which are present almost exclusively in obligate anaerobic or microaerophilic microorganisms [22,23,24,25,26,27,28,29,30,31,32,33,34,35,36,37,38,39,40,41,42,43,44,45,46,47,48,49,50,51,52,53,54,55,56,57,58,59]. Once reduced, metronidazole generates highly reactive nitro radicals that induce DNA single- and double-strand breaks, loss of genomic integrity, inhibition of nucleic acid synthesis, and cell death. In the absence of redox activation systems, the drug remains inactive and is eliminated without exerting antimicrobial effects.

#### 4.4.2. Probiotics Biologically Compatible with Antibiotics Active on Anaerobic Metabolism

Microorganisms lacking the redox systems required for nitroimidazole activation are intrinsically insensitive to this class of antibiotics. This condition does not represent resistance but rather the absence of a functional metabolic target. It includes lactobacilli, bifidobacteria, spore-forming bacteria, and probiotic yeasts.

**Probiotic Yeasts**

*S. boulardii* lacks the redox systems for the activation of nitroimidazoles, does not present antibacterial molecular targets, and is not affected by antibiotics that affect anaerobic metabolism [58,59].

**Spore-Forming Bacteria**

Spore-forming probiotics such as *B. clausii*, *C. butyricum*, and *H. coagulans* exhibit intrinsic insensitivity during sporulation or metabolic quiescence, when redox activity and transcriptional processes are minimal. Under these conditions, metronidazole cannot be activated and therefore lacks an effective antimicrobial target [1,2,3,4,5,6,7,8,9,10,11,12,13,14,15,16,17,18,19,20,21,22,23,24,25,26,27,28,29,30,31,32,33,34,35,36,37,38,39,40,41,42,43,44,45].

**Aerotolerant or Microaerophilic Bacteria**

Lactobacilli are aerotolerant or microaerophilic bacteria, characterized by a predominantly fermentative metabolism. They lack low-redox ferredoxins and nitroreductases capable of reducing the nitro group of metronidazole and do not generate the intracellular redox environment required for drug activation. Consequently, metronidazole remains inactive within the cell, without generating cytotoxic radicals or DNA damage [50]. In vitro susceptibility studies consistently show high MICs or absence of growth inhibition for several *Lactobacillus* species (including *L. rhamnosus*, *L. plantarum*, *L. reuteri*, *L. acidophilus*), confirming an intrinsic and non-transferable resistance [47,48,49,50,51,52,53,54,55,56,57,58,59,60,61,62].

**Obligate Anaerobes**

Although bifidobacteria are obligate anaerobes, their metabolic organization differs substantially from that of metronidazole-sensitive anaerobes such as *Bacteroides* or *Clostridioides*. Their energy metabolism relies on the fructose-6-phosphate phosphoketolase pathway (bifido shunt), which does not involve ferredoxins or nitroreductases required for nitroimidazole activation. As a result, the intracellular conditions do not favor nitro group reduction. Systematic susceptibility studies show that *Bifidobacterium* species (*B. longum*, *B. breve*, *B. adolescentis*, and *B. bifidum*) are intrinsically resistant or poorly sensitive to metronidazole, with high MICs and no bactericidal activity [45,46,47,48,49,50,51,52,53,54,55,56,57,58,59,60,61,62]. This insensitivity is metabolic rather than genetic and is particularly relevant in vivo, where bifidobacteria can further enhance persistence through slowed metabolism and adaptive stress response during antibiotic exposure [53].

#### 4.4.3. Conceptual Synthesis

**Intrinsic Resistance**
  - **Absence of the target:** *S. boulardii*.
  - **Absence of drug activation systems:**

Spore-forming probiotics (*B. clausii*, *C. butyricum* and *H. coagulans*).

*Lactobacillus* species (*L. rhamnosus*, *L. plantarum*, *L. reuteri*, *L. acidophilus*).

*Bifidobacterium* species (*B. longum*, *B. breve*, *B. adolescentis*, and *B. bifidum*).

An overview of probiotic biological compatibility with antibiotics active on anaerobic metabolism is presented in Figure 1.

## 5. Biological and Ecological Rules Governing Probiotic-Antibiotic Interactions

From an ecological perspective, antibiotics exert selective forces that reshape microbial niches rather than acting as purely antimicrobial agents. Probiotics are given to correct antibiotic dysbiosis. However, probiotics administration is not yet based on a substantial biological rationale that would guarantee probiotic survival and functionality during antibiotic exposure.

Our work supports the notion that probiotic compatibility with antibiotics extends beyond classical resistance profiles and reflects biological traits such as metabolic activity, stress adaptation, and ecological niche exploitation following antibiotic-induced microbiota disruption. Antibiotics targeting nucleic acid synthesis mainly affect metabolically active bacteria, while quiescent or spore-forming microorganisms remain less susceptible, indicating that susceptibility depends not only on target presence but also on permeability, intracellular accumulation, and metabolic state [10]. Such traits can confer intrinsic antibiotic compatibility without transferable resistance determinants: spore-forming bacteria evade antibiotics through dormancy, while probiotic yeasts, as eukaryotes, are inherently unaffected by antibacterial agents, supporting their biological safety.

Finally, another aspect that should be taken into consideration is the co-administration of broad-spectrum antibiotics [63]. This group includes combinations of β-lactams/β-lactamase inhibitors and carbapenems combined with specific enzyme inhibitors: amoxicillin-clavulanate and ampicillin-sulbactam [25]; ureidopenicillins with inhibitors, particularly piperacillin/tazobactam [64]; ceftolozane + tazobactam and ceftazidime + avibactam; meropenem + vaborbactam. In the context of broad-spectrum therapies, predicting the compatibility of specific probiotic or commensal strains becomes particularly complex and depends on the integration of multiple physiological and ecological factors [65], such as metabolic state (active vs. quiescent), sporulation capacity, cell wall architecture and permeability, presence of chromosomal β-lactamases, ecological resilience, and recolonization capacity.

### 5.1. Biofilm Formation as an Ecological Determinant of Probiotic-Antibiotic Interaction

Another way that we haven’t discussed so far is how probiotics could survive antibiotic treatment, which is biofilm formation. This strategy consists of the synthesis of an extracellular matrix that hinders the diffusion of antibiotic compounds [64,65].

Biofilm-producing strains with potential probiotic activity include mostly strains of the genus *Lactobacillus. L. reuteri* is a well-established example, forming metabolically active biofilms capable of sustained production of antimicrobial and immunomodulatory compounds, with enhanced resilience to environmental stressors [66]. Similarly, *L. rhamnosus* GG exhibits pili-mediated adhesion and microcolony formation, contributing to persistence and functional activity at the mucosal surface [67]. Importantly, in lactobacilli, this phenotype is strongly strain-dependent and cannot be generalized at the species or genus level [68].

Evidence is also emerging for biofilm formation in specific *Bifidobacterium* strains, including *B. breve*, *B. longum*, and *B. adolescentis*, particularly under gut-relevant conditions such as bile exposure or mixed-species communities. In these contexts, biofilm growth has been associated with increased persistence, extracellular polymeric matrix production, and enhanced ecological fitness rather than classical antimicrobial resistance [67,68].

In contrast, oral probiotics such as *Streptococcus salivarius* K12 and M18 play a distinct ecological role. These strains are not primarily characterized by robust self-produced biofilm formation, but rather by their capacity to integrate into commensal oral biofilms and modulate their structure, exerting antagonistic effects against pathogenic biofilm-forming species such as *Streptococcus mutans* through bacteriocin production and interference with biofilm development [69]. Their relevance, therefore, lies in biofilm modulation rather than biofilm-mediated protection from an ecological and experimental perspective.

Notably, the contribution of biofilm formation to reduced antibiotic susceptibility is time-dependent. This means that antibiotic tolerance is more likely to occur during prophylactic or early probiotic administration than during ongoing antibiotic exposure. Therefore, probiotic–antibiotic compatibility depends not only on biofilm formation itself but also on timing and spatial organization within antibiotic-perturbed ecosystems.

### 5.2. Role of Intestinal Antibiotic Exposure and Timing

Another factor that should be considered in probiotic-antibiotic interactions is represented by a diverse drug exposure of the gut microbiota commensals. Following systemic administration, antibiotic concentrations within the intestinal lumen are neither uniform nor predictable, as drug availability depends on multiple pharmacokinetic variables, including the site and extent of absorption, oral bioavailability, biliary excretion, and intestinal transit. The ecological impact on the gut microbiota is therefore shaped not only by antimicrobial activity *per se*, but also by the spatial distribution, composition, and density of microbial communities along the gastrointestinal tract [69,70]. Consequently, antibiotic-induced dysbiosis reflects both antimicrobial efficacy and substantial inter-individual variability in luminal exposure and local ecological factors that modulate microbial susceptibility and resilience [8]. Consequently, gut microorganisms are exposed to highly heterogeneous and often subinhibitory antibiotic concentrations, both spatially and temporally, which may promote functional insensitivity, persistence, or stress-adaptive responses rather than outright eradication [71,72].

Timing further modulates this interaction: early antibiotic exposure primarily affects the metabolically active luminal population, whereas later phases preferentially shape recolonization dynamics and niche availability. In this context, probiotic survival and functionality may depend less on absolute resistance profiles and more on the ability to persist or exploit ecological windows created by fluctuating antibiotic pressure [73,74].

Integrating intestinal antibiotic pharmacokinetics and pharmacodynamics with microbial physiology, therefore, represents a critical step toward a biologically coherent interpretation of probiotic compatibility during antibiotic therapy.

## 6. Limitations and Open Questions

The proposed conceptual framework provides a biologically plausible interpretation of antibiotic-probiotic interactions; however, several limitations must be acknowledged to avoid overinterpretation and to define priorities for future research.

This work is primarily conceptual, and the proposed framework is based on known antibiotic mechanisms and microbial has not yet been systematically validated experimentally or clinically.

There are several critical aspects and experimental limitations related to the current state of knowledge on bacterial interactions.

Microbial metabolic state and quiescence, which resulted in being an important pillar of probiotic compatibility to antibiotics, are highly dynamic and remain difficult to assess in vivo. The use of DNA-based profiling as an indicator of bacterial diversity and a predictive tool of bacterial interactions does not discriminate between active and quiescent cells and still presents numerous limitations in terms of standardization, resolution, and clinical feasibility [13]. Biological compatibility should be addressed at the strain level, as functional insensitivity to antibiotics can vary substantially even within the same species. In fact, strain-specific traits can determine whether a microorganism remains functionally unaffected under antibiotic pressure. Therefore, assessing compatibility at higher taxonomic levels may overlook important functional differences that are critical for predicting probiotic performance during antibiotic therapy [13,14,15,16,17,18,19,20].

Antibiotic-microbiota interactions may remain inherently simplified. On the one hand, antibiotics act not only through direct antimicrobial effects but also by affecting microbial interactions and host-microbiota dynamics [5,22,23,24,25,26,27,28,29,30,31,32,33,34,35,36,37,38,39,40,41,42,43,44,45,46,47,48,49,50,51,52,53,54,55,56,57,58,59,60,61,62,63,64,65,66,67,68,69,70,71,72,73,74,75,76,77,78]. On the other hand, microbiota composition, and thus responses to both antibiotics and probiotics, can be shaped by individual and environmental factors [15]. These factors limit the applicability of future studies which should aim to better capture the complexity of host-microbial ecosystems, potentially through personalized medicine strategies.

Finally, the concept of probiotics’ biological compatibility with antibiotic classes must be validated to determine whether it leads to measurable functional or clinical benefits.

## 7. Safety Considerations on Probiotics Use During Antibiotic Therapy

The safety evaluation of probiotics administered during antibiotic therapy must consider both host-related safety aspects and microbiological risks related to antimicrobial resistance.

Although probiotics are generally considered safe, rare cases of probiotic-associated infections, including bacteremia or fungemia, have been reported, particularly in severely immunocompromised individuals, critically ill patients, or patients with central venous catheters [79,80]. For this reason, an appropriate evaluation of the clinical history of the patient remains essential, especially for vulnerable populations.

A critical, yet often underestimated, aspect concerns the potential role of probiotics in the context of antimicrobial resistance (AR) and AR genes (ARGs). International recommendations emphasize the importance of genomic characterization and exclusion of transferable ARGs in probiotic strains intended for clinical use [79,80,81].

In theory, even strains initially lacking ARGs may acquire them through horizontal gene transfer in the gut environment, potentially expanding the individual’s gut reservoir of resistance genes [82,83]. This event depends initially on strain-specific genetic traits, the abundance of ARG-harboring bacteria, and selective pressures such as concurrent antibiotic therapy [79,80,81,82,83,84].

Although the likelihood that probiotic strains acquire antimicrobial resistance genes (ARGs) and become harmful appears low, this risk cannot be negligible, particularly in individuals with disrupted microbiota or prolonged antibiotic exposure.

Our final consideration is that safety of probiotics should be evaluated within a personalized medicine framework, especially because host-related factors, such as immune response, mucosal inflammation, and individual antibiotic pharmacokinetics, are increasingly recognized as key modulators of both antibiotic effects on the microbiota and probiotic functionality [16].

## 8. Future Prospects

Increasing evidence shows that antibiotics act as ecological modifiers of the gut microbiota. This perspective challenges empirical probiotic selection during antibiotic therapy and supports the development of biologically informed frameworks to guide future research and rational probiotic design. Here, we outline several research directions that may help advance this field.

A priority is the experimental validation of the concept of biological compatibility. In vitro and ex vivo models, including a dynamic fermentation system, could systematically explore probiotic viability, metabolic activity, and functional output in the presence of specific antibiotic classes, clearly distinguishing between genetic resistance and functional insensitivity related to the metabolic or structural state of the microorganism [1,2,3,4,5,6,7,8,9,10,11,12,13].

Animal and gnotobiotic models represent a complementary line of investigation, allowing controlled analysis of antibiotic-probiotic-microbiota interactions and the specific contribution of metabolic quiescence, persistence, and ecological niche occupation to strain survival and functionality [22]. These approaches would help clarify ecosystem-level effects, such as SCFAs production, colonization resistance, and the stability of microbial networks.

From a clinical perspective, future studies should prioritize antibiotic-probiotic coadministration trials stratified by antibiotic class rather than by individual probiotic strains. This approach could reduce heterogeneity across patients and enable more consistent evaluation of microbiological and clinical outcomes. [10].

An additional area of interest for future research is to assess safety regarding antibiotic resistance. Even probiotic strains initially devoid of transferable resistance genes may, under selective pressure, acquire ARGs [80,81]. Integrating genomic surveillance, metagenomic monitoring, and careful strain selection might minimize AR risk [79,80,81,82,83,84,85].

On the other hand, new bioengineered probiotics could be formulated with strains capable of maintaining functionality under antibiotic pressure and at the same time refractory to horizontal ARG acquisition [2,3,4,5,6,7,8,9,10,11,12,13,14,15,16,17,18,19,20].

Integration of functional microbiomics with metabolomics could further enable personalized microbiota-support strategies [2,3,4,5,6,7,8,9,10,11,12,13,14,15,16,17,18,19,20,21,22,23,24,25,26,27,28,29,30,31,32,33,34,35,36,37,38,39,40,41,42,43,44,45,46,47,48,49,50,51,52,53,54,55,56,57,58,59,60,61,62,63,64,65,66,67,68,69,70,71,72,73,74,75,76,77,78,79,80,81,82,83,84,85,86,87,88,89,90,91,92].

## 9. Conclusions

The use of probiotics during antibiotic therapy is widespread in clinical practice, but remains largely guided by an empirical approach, often based on aggregate evidence of efficacy rather than an explicit biological rationale. This conceptual review proposes a shift in perspective: considering antibiotic-probiotic combinations as an ecological interaction between selective pressure and a microorganism with specific structural, genomic, metabolic, and functional characteristics.

By analyzing the mechanisms of action of antibiotics, it emerges that the survival and functionality of probiotics do not depend exclusively on the presence of genetic determinants of resistance. Conversely, factors such as metabolic state, quiescence, cell wall structure, membrane permeability, and drug activation capacity play a central role in determining functional insensitivity to antibiotics, a concept distinct from and not overlapping with acquired resistance [13].

Importantly, even strains lacking transferable antibiotic resistance genes may, under selective pressures in the gut, acquire and potentially disseminate AR determinants via horizontal gene transfer [80,81,82,83,84,85,86,87,88,89]. Probiotic use, therefore, requires careful strain selection, genomic characterization, and consideration of recipient microbiota dynamics [79,80,81,82,83,84,85]

In conclusion, shifting attention from simple “strain efficacy” to the ecological coherence of the intervention represents a necessary step towards a more rational and scientifically sound use of probiotics during antibiotic therapy. Adopting this paradigm could, over time, contribute to reducing the collateral impact of antibiotics on the microbiota and promoting safer, more targeted, and biologically sustainable microbial support interventions.

## Figures and Tables

**Table 1:** Definition of antibiotic resistance, tolerance, persistence, and functional insensitivity relevant to probiotic–antibiotic compatibility.

Bacterial Response to Antibiotic Exposure	MIC	Bacterial Killing Dynamics	Biological Mechanism	Relevance for Probiotic–Antibiotic Compatibility
Resistance	Increased	Bacterial killing reduced or absent	Genetic determinants such as target modification, drug inactivation, or efflux pumps	May raise safety concerns if resistance genes are transferable
Tolerance	Unchanged	Slower bacterial killing despite susceptibility	Physiological adaptations affecting antibiotic action (e.g., stress responses, metabolic state)	No long-term colonization
Persistence	Unchanged	Small subpopulation survives prolonged antibiotic exposure	Persister cell formation, metabolic dormancy, stress-response activation, and biofilm-associated protection	No long-term colonization
Functional insensitivity	Typically unchanged	Minimal or absent antibiotic effect due to lack of effective interaction with cellular targets	Absence or inaccessibility of antibiotic target, structural barriers, physiological traits limiting antibiotic activity	May allow probiotic survival without involving resistance mechanisms

**Figure A1:**
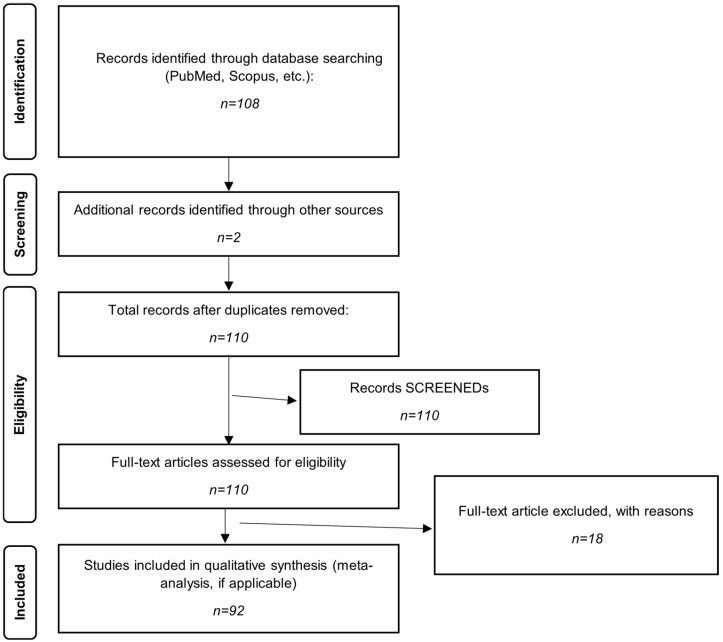
A flow diagram of the search process.

## Data Availability

No new data were created or analyzed in this study. Data sharing is not applicable to this article.

## References

[1] Neut C., Mahieux S., Dubreuil L.J. (2017). Antibiotic susceptibility of probiotic strains: Is it reasonable to combine probiotics with antibiotics?. Méd. Mal. Infect..

[2] Gueimonde M., Sánchez B., de los Reyes-Gavilán C.G., Margolles A. (2013). Antibiotic resistance in probiotic bacteria. Front. Microbiol..

[3] Benoun J.M., Labuda J.C., McSorley S.J. (2016). Collateral Damage: Detrimental Effect of Antibiotics on the Development of Protective Immune Memory. mBio.

[4] Narikawa S. (1986). Distribution of metronidazole susceptibility factors in obligate anaerobes. J. Antimicrob. Chemother..

[5] Ianiro G., Tilg H., Gasbarrini A. (2016). Antibiotics as deep modulators of gut microbiota: Between good and evil. Gut.

[6] Palleja A., Mikkelsen K.H., Forslund S.K., Kashani A., Allin K.H., Nielsen T., Hansen T.H., Liang S., Feng Q., Zhang C. (2018). Recovery of gut microbiota of healthy adults following antibiotic exposure. Nat. Microbiol..

[7] Dethlefsen L., Huse S., Sogin M.L., Relman D.A. (2008). The Pervasive Effects of an Antibiotic on the Human Gut Microbiota, as Revealed by Deep 16S rRNA Sequencing. PLoS Biol..

[8] Guarner F., Bustos Fernandez L., Cruchet S., Damião A., Maruy Saito A., Riveros Lopez J.P., Rodrigues Silva L., Valdovinos Diaz M.A. (2024). Gut dysbiosis mediates the association between antibiotic exposure and chronic disease. Front. Med..

[9] Props R., Kerckhof F.-M., Rubbens P., De Vrieze J., Sanabria E.H., Waegeman W., Monsieurs P., Hammes F., Boon N. (2017). Absolute quantification of microbial taxon abundances. ISME J..

[10] Zimmermann P., Curtis N. (2019). The effect of antibiotics on the composition of the intestinal microbiota—A systematic review. J. Infect..

[11] Manyemba J., Mayosi B.M. (2002). Penicillin for secondary prevention of rheumatic fever. Cochrane Database Syst. Rev..

[12] Becattini S., Taur Y., Pamer E.G. (2016). Antibiotic-Induced Changes in the Intestinal Microbiota and Disease. Trends Mol. Med..

[13] Brauner A., Fridman O., Gefen O., Balaban N.Q. (2016). Distinguishing between resistance, tolerance and persistence to antibiotic treatment. Nat. Rev. Microbiol..

[14] Koutsoumanis K., Allende A., Alvarez-Ordóñez A., Bolton D., Bover-Cid S., Chemaly M., Davies R., De Cesare A., Hilbert F., EFSA Panel on Biological Hazards (BIOHAZ) (2022). Update of the list of QPS-recommended biological agents intentionally added to food or feed as notified to EFSA 15: Suitability of taxonomic units notified to EFSA until September 2021. EFSA J..

[15] Hill C., Guarner F., Reid G., Gibson G.R., Merenstein D.J., Pot B., Morelli L., Canani R.B., Flint H.J., Salminen S. (2014). The International Scientific Association for Probiotics and Prebiotics consensus statement on the scope and appropriate use of the term probiotic. Nat. Rev. Gastroenterol. Hepatol..

[16] Yang S., Qiao J., Zhang M., Kwok L.-Y., Matijašić B.B., Zhang H., Zhang W. (2025). Prevention and treatment of antibiotics-associated adverse effects through the use of probiotics: A review. J. Adv. Res..

[17] Campedelli I., Mathur H., Salvetti E., Clarke S., Rea M.C., Torriani S., Ross R.P., Hill C., O’Toole P.W. (2019). Genus-Wide Assessment of Antibiotic Resistance in *Lactobacillus* spp.. Appl. Environ. Microbiol..

[18] Mangin I., Lévêque C., Magne F., Suau A., Pochart P. (2012). Long-Term Changes in Human Colonic *Bifidobacterium* Populations Induced by a 5-Day Oral Amoxicillin-Clavulanic Acid Treatment. PLoS ONE.

[19] Koch A.L. (2003). Bacterial Wall as Target for Attack. Clin. Microbiol. Rev..

[20] Bush K., Bradford P.A. (2016). β-Lactams and β-Lactamase Inhibitors: An Overview. Cold Spring Harb. Perspect. Med..

[21] Tuomanen E., Cozens R., Tosch W., Zak O., Tomasz A. (1986). The Rate of Killing of Escherichia coli by -Lactam Antibiotics Is Strictly Proportional to the Rate of Bacterial Growth. Microbiology.

[22] Kohanski M.A., Dwyer D.J., Collins J.J. (2010). How antibiotics kill bacteria: From targets to networks. Nat. Rev. Microbiol..

[23] Perkins H.R., Nieto M. (1974). THE CHEMICAL BASIS FOR THE ACTION OF THE VANCOMYCIN GROUP OF ANTIBIOTICS. Ann. N. Y. Acad. Sci..

[24] Reynolds P.E. (1989). Structure, biochemistry and mechanism of action of glycopeptide antibiotics. Eur. J. Clin. Microbiol. Infect. Dis..

[25] Bush K. (2018). Past and Present Perspectives on β-Lactamases. Antimicrob. Agents Chemother..

[26] Nikaido H. (2003). Molecular Basis of Bacterial Outer Membrane Permeability Revisited. Microbiol. Mol. Biol. Rev..

[27] Delcour A.H. (2009). Outer membrane permeability and antibiotic resistance. Biochim. Biophys. Acta (BBA)-Proteins Proteom..

[28] Pagès J.-M., James C.E., Winterhalter M. (2008). The porin and the permeating antibiotic: A selective diffusion barrier in Gram-negative bacteria. Nat. Rev. Microbiol..

[29] Li X.-Z., Plésiat P., Nikaido H. (2015). The Challenge of Efflux-Mediated Antibiotic Resistance in Gram-Negative Bacteria. Clin. Microbiol. Rev..

[30] Schleifer K.H., Kandler O. (1972). Peptidoglycan types of bacterial cell walls and their taxonomic implications. Bacteriol. Rev..

[31] Ramos-Vivas J. (2020). Microbiología de Hafnia alvei. Enferm. Infecc. Microbiol. Clín..

[32] Kaźmierczak-Siedlecka K., Ruszkowski J., Fic M., Folwarski M., Makarewicz W. (2020). *Saccharomyces boulardii* CNCM I-745: A Non-bacterial Microorganism Used as Probiotic Agent in Supporting Treatment of Selected Diseases. Curr. Microbiol..

[33] Mancabelli L., Mancino W., Lugli G.A., Argentini C., Longhi G., Milani C., Viappiani A., Anzalone R., Bernasconi S., van Sinderen D. (2021). Amoxicillin-Clavulanic Acid Resistance in the Genus *Bifidobacterium*. Appl. Environ. Microbiol..

[34] Di Pierro F., Campedelli I., De Marta P., Fracchetti F., Del Casale A., Cavecchia I., Matera M., Cazzaniga M., Bertuccioli A., Guasti L. (2023). *Bifidobacterium breve* PRL2020: Antibiotic-Resistant Profile and Genomic Detection of Antibiotic Resistance Determinants. Microorganisms.

[35] Deghorain M., Goffin P., Fontaine L., Mainardi J.-L., Daniel R., Errington J., Hallet B., Hols P. (2007). Selectivity for D-Lactate Incorporation into the Peptidoglycan Precursors of *Lactobacillus plantarum*: Role of Aad, a VanX-Like D-Alanyl-D-Alanine Dipeptidase. J. Bacteriol..

[36] Wilson D.N. (2014). Ribosome-targeting antibiotics and mechanisms of bacterial resistance. Nat. Rev. Microbiol..

[37] Stoeva M.K., Garcia-So J., Justice N., Myers J., Tyagi S., Nemchek M., McMurdie P.J., Kolterman O., Eid J. (2021). Butyrate-producing human gut symbiont, *Clostridium butyricum*, and its role in health and disease. Gut Microbes.

[38] Bryan L.E., Kwan S. (1981). Mechanisms of aminoglycoside resistance of anaerobic bacteria and facultative bacteria grown anaerobically. J. Antimicrob. Chemother..

[39] Taber H.W., Mueller J.P., Miller P.F., Arrow A.S. (1987). Bacterial uptake of aminoglycoside antibiotics. Microbiol. Rev..

[40] Tereshchenkov A.G., Dobosz-Bartoszek M., Osterman I.A., Marks J., Sergeeva V.A., Kasatsky P., Komarova (Andreyanova) E.S., Stavrianidi A.N., Rodin I.A., Konevega A.L. (2018). Binding and Action of Amino Acid Analogs of Chloramphenicol upon the Bacterial Ribosome. J. Mol. Biol..

[41] Schlünzen F., Zarivach R., Harms J., Bashan A., Tocilj A., Albrecht R., Yonath A., Franceschi F. (2001). Structural basis for the interaction of antibiotics with the peptidyl transferase centre in eubacteria. Nature.

[42] Vazquez D. (1974). Inhibitors of protein synthesis. FEBS Lett..

[43] Urdaci M.C., Bressollier P., Pinchuk I. (2004). *Bacillus clausii* Probiotic Strains. J. Clin. Gastroenterol..

[44] Chopra I., Roberts M. (2001). Tetracycline Antibiotics: Mode of Action, Applications, Molecular Biology, and Epidemiology of Bacterial Resistance. Microbiol. Mol. Biol. Rev..

[45] Moubareck C., Gavini F., Vaugien L., Butel M.J., Doucet-Populaire F. (2005). Antimicrobial susceptibility of bifidobacteria. J. Antimicrob. Chemother..

[46] Drago L., Rodighiero V., Mattina R., Toscano M., de Vecchi E. (2011). In Vitro Selection and Transferability of Antibiotic Resistance in the Probiotic Strain *Lactobacillus reuteri* DSM 17938. J. Chemother..

[47] Danielsen M., Wind A. (2003). Susceptibility of *Lactobacillus* spp. to antimicrobial agents. Int. J. Food Microbiol..

[48] Drlica K., Zhao X. (1997). DNA gyrase, topoisomerase IV, and the 4-quinolones. Microbiol. Mol. Biol. Rev..

[49] Yu X., Jin Y., Zhou W., Xiao T., Wu Z., Su J., Gao H., Shen P., Zheng B., Luo Q. (2022). Rifaximin Modulates the Gut Microbiota to Prevent Hepatic Encephalopathy in Liver Cirrhosis Without Impacting the Resistome. Front. Cell. Infect. Microbiol..

[50] Edwards D.I. (1993). Nitroimidazole drugs-action and resistance mechanisms I. Mechanism of action. J. Antimicrob. Chemother..

[51] Wood T.K., Knabel S.J., Kwan B.W. (2013). Bacterial Persister Cell Formation and Dormancy. Appl. Environ. Microbiol..

[52] Ghelardi E., Abreu y Abreu A.T., Marzet C.B., Álvarez Calatayud G., Perez M., Moschione Castro A.P. (2022). Current Progress and Future Perspectives on the Use of *Bacillus clausii*. Microorganisms.

[53] Schöpping M., Zeidan A.A., Franzén C.J. (2022). Stress Response in Bifidobacteria. Microbiol. Mol. Biol. Rev..

[54] Di Pierro F., Pane M. (2021). *Bifidobacterium longum* W11: Uniqueness and individual or combined clinical use in association with rifaximin. Clin. Nutr. ESPEN.

[55] Isa K., Oka K., Beauchamp N., Sato M., Wada K., Ohtani K., Nakanishi S., McCartney E., Tanaka M., Shimizu T. (2016). Safety assessment of the *Clostridium butyricum* MIYAIRI 588^®^ probiotic strain including evaluation of antimicrobial sensitivity and presence of *Clostridium* toxin genes in vitro and teratogenicity in vivo. Hum. Exp. Toxicol..

[56] Graziano T., Amoruso A., Nicola S., Deidda F., Allesina S., Pane M., Piffanelli P., Strozzi F., Mogna L., Del Piano M. (2016). The Possible Innovative Use of *Bifidobacterium longum* W11 in Association with Rifaximin. J. Clin. Gastroenterol..

[57] Merenstein D., Fraser C.M., Roberts R.F., Liu T., Grant-Beurmann S., Tan T.P., Smith K.H., Cronin T., Martin O.A., Sanders M.E. (2021). *Bifidobacterium animalis* subsp. *lactis* BB-12 Protects against Antibiotic-Induced Functional and Compositional Changes in Human Fecal Microbiome. Nutrients.

[58] Grozdanov L., Zähringer U., Blum-Oehler G., Brade L., Henne A., Knirel Y.A., Schombel U., Schulze J., Sonnenborn U., Gottschalk G. (2002). A Single Nucleotide Exchange in the *wzy* Gene Is Responsible for the Semirough O6 Lipopolysaccharide Phenotype and Serum Sensitivity of *Escherichia coli* Strain Nissle 1917. J. Bacteriol..

[59] Leitsch D. (2019). A review on metronidazole: An old warhorse in antimicrobial chemotherapy. Parasitology.

[60] Szajewska H., Kołodziej M., Skórka A., Pieścik-Lech M. (2022). Infant Formulas with Postbiotics. J. Pediatr. Gastroenterol. Nutr..

[61] McFarland L.V. (2010). Systematic review and meta-analysis of *Saccharomyces boulardii* in adult patients. World J. Gastroenterol..

[62] Charteris W.P., Kelly P.M., Morelli L., Collins J.K. (1998). Antibiotic Susceptibility of Potentially Probiotic *Lactobacillus* Species. J. Food Prot..

[63] Leekha S., Terrell C.L., Edson R.S. (2011). General Principles of Antimicrobial Therapy. Mayo Clin. Proc..

[64] Drawz S.M., Bonomo R.A. (2010). Three Decades of β-Lactamase Inhibitors. Clin. Microbiol. Rev..

[65] Konings W.N., Lolkema J.S., Bolhuis H., van Veen H.W., Poolman B., Driessen A.J.M. (1997). The role of transport processes in survival of lactic acid bacteria, Energy transduction and multidrug resistance. Antonie van Leeuwenhoek.

[66] Jones S.E., Versalovic J. (2009). Probiotic *Lactobacillus reuteri* biofilms produce antimicrobial and anti-inflammatory factors. BMC Microbiol..

[67] Lebeer S., Verhoeven T.L., Perea Vélez M., Vanderleyden J., De Keersmaecker S.C. (2007). Impact of Environmental and Genetic Factors on Biofilm Formation by the Probiotic Strain *Lactobacillus rhamnosus* GG. Appl. Environ. Microbiol..

[68] Salas-Jara M.J., Ilabaca A., Vega M., García A. (2016). Biofilm Forming *Lactobacillus*: New Challenges for the Development of Probiotics. Microorganisms.

[69] Reichardt E., Shyp V., Alig L., Verna C., Kulik E.M., Bornstein M.M. (2024). Antimicrobial effect of probiotic bacteriocins on *Streptococcus mutans* biofilm in a dynamic oral flow chamber model—An in vitro study. J. Oral Microbiol..

[70] Xu T., Xiao Y., Wang H., Zhu J., Lee Y., Zhao J., Lu W., Zhang H. (2022). Characterization of Mixed-Species Biofilms Formed by Four Gut Microbiota. Microorganisms.

[71] Pilmis B., Le Monnier A., Zahar J.-R. (2020). Gut Microbiota, Antibiotic Therapy and Antimicrobial Resistance: A Narrative Review. Microorganisms.

[72] Maier L., Goemans C.V., Wirbel J., Kuhn M., Eberl C., Pruteanu M., Müller P., Garcia-Santamarina S., Cacace E., Zhang B. (2021). Unravelling the collateral damage of antibiotics on gut bacteria. Nature.

[73] Andersson D.I., Hughes D. (2014). Microbiological effects of sublethal levels of antibiotics. Nat. Rev. Microbiol..

[74] Pamer E.G. (2016). Resurrecting the intestinal microbiota to combat antibiotic-resistant pathogens. Science.

[75] Flemming H.-C., Wingender J., Szewzyk U., Steinberg P., Rice S.A., Kjelleberg S. (2016). Biofilms: An emergent form of bacterial life. Nat. Rev. Microbiol..

[76] Costerton J.W., Stewart P.S., Greenberg E.P. (1999). Bacterial Biofilms: A Common Cause of Persistent Infections. Science.

[77] Kelly S.M., Lanigan N., O’Neill I.J., Bottacini F., Lugli G.A., Viappiani A., Turroni F., Ventura M., van Sinderen D. (2020). Bifidobacterial biofilm formation is a multifactorial adaptive phenomenon in response to bile exposure. Sci. Rep..

[78] Schwartz D.J., Langdon A.E., Dantas G. (2020). Understanding the impact of antibiotic perturbation on the human microbiome. Genome Med..

[79] Sanders M.E., Akkermans L.M.A., Haller D., Hammerman C., Heimbach J.T., Hörmannsperger G., Huys G. (2010). Safety assessment of probiotics for human use. Gut Microbes.

[80] Didari T. (2015). Effectiveness of probiotics in irritable bowel syndrome: Updated systematic review with meta-analysis. World J. Gastroenterol..

[81] (2012). EFSA Panel on Additives and Products or Substances used in Animal Feed (FEEDAP). Guidance on the assessment of bacterial susceptibility to antimicrobials of human and veterinary importance. EFSA J..

[82] van Schaik W. (2015). The human gut resistome. Philos. Trans. R. Soc. B Biol. Sci..

[83] Mathur S., Singh R. (2005). Antibiotic resistance in food lactic acid bacteria—A review. Int. J. Food Microbiol..

[84] Dogra S.K., Doré J., Damak S. (2020). Gut Microbiota Resilience: Definition, Link to Health and Strategies for Intervention. Front. Microbiol..

[85] Zheng M., Zhang R., Tian X., Zhou X., Pan X., Wong A. (2017). Assessing the Risk of Probiotic Dietary Supplements in the Context of Antibiotic Resistance. Front. Microbiol..

[86] Montassier E., Valdés-Mas R., Batard E., Zmora N., Dori-Bachash M., Suez J., Elinav E. (2021). Probiotics impact the antibiotic resistance gene reservoir along the human GI tract in a person-specific and antibiotic-dependent manner. Nat. Microbiol..

[87] Rychen G., Aquilina G., Azimonti G., Bampidis V., de Lourdes Bastos M., Bories G., Chesson A., Cocconcelli P.S., Flachowsky G., EFSA Panel on Additives and Products or Substances Used in Animal Feed (FEEDAP) (2018). Guidance on the characterisation of microorganisms used as feed additives or as production organisms. EFSA J..

[88] Zheng J., Wittouck S., Salvetti E., Franz C.M.A.P., Harris H.M.B., Mattarelli P., O’Toole P.W., Pot B., Vandamme P., Walter J. (2020). A taxonomic note on the genus *Lactobacillus*: Description of 23 novel genera, emended description of the genus Lactobacillus Beijerinck 1901, and union of *Lactobacillaceae* and *Leuconostocaceae*. Int. J. Syst. Evol. Microbiol..

[89] Imperial I.C.V.J., Ibana J.A. (2016). Addressing the Antibiotic Resistance Problem with Probiotics: Reducing the Risk of Its Double-Edged Sword Effect. Front. Microbiol..

[90] Allende A., Alvarez-Ordóñez A., Bortolaia V., Bover-Cid S., De Cesare A., Dohmen W., Guillier L., Jacxsens L., Nauta M., EFSA Panel on Biological Hazards (BIOHAZ) (2026). Update of the list of QPS-recommended biological agents intentionally added to food or feeds as notified to EFSA. EFSA J..

[91] Salvetti E., Torriani S., Felis G.E. (2012). The Genus *Lactobacillus*: A Taxonomic Update. Probiotics Antimicrob. Proteins.

[92] van Reenen C.A., Dicks L.M.T. (2011). Horizontal gene transfer amongst probiotic lactic acid bacteria and other intestinal microbiota: What are the possibilities? A review. Arch. Microbiol..

